# „Die denken immer, man ist ein Killer“ – Reaktionsweisen von Schlachthofarbeitern auf moralische Stigmatisierung

**DOI:** 10.1007/s11614-021-00450-3

**Published:** 2021-07-22

**Authors:** Marcel Sebastian

**Affiliations:** Fachbereich Sozialwissenschaften, Universität Hamburg, Allende-Platz 1, 20146 Hamburg, Deutschland

**Keywords:** Stigma, Schlachthofarbeit, Kultur, Mensch-Tier-Beziehung, Dirty work, Stigma, Slaughterhouse work, Culture, Human-animal relations, Dirty work

## Abstract

Schlachthofarbeit hat einen schlechten Ruf und viele Schlachter erfahren moralische Stigmatisierung, obwohl Fleischkonsum die dominante Ernährungsform westlicher Gesellschaften ist. Moralische Stigmatisierung von Schlachthofarbeitern wurde bisher nicht systematisch untersucht. Der Artikel beantwortet die Forschungsfragen, welche Strategien des Umgangs mit moralischer Stigmatisierung sich unter Schlachthofarbeitern finden und wie sie sich zu hegemonialen Erzählungen zu ihrem Beruf verhalten. Der theoretische Rahmen des Artikels basiert auf soziologischen Theorien zu Kultur, Stigma und *dirty work*. Zur Beantwortung der Forschungsfragen wurden 13 problemzentrierte Interviews mit Arbeitern aus sechs deutschen Schlachthöfen mittels Qualitativer Inhaltsanalyse ausgewertet. Die Analyse ergab, dass Schlachter auf moralische Stigmatisierung reagieren, indem sie diejenigen kulturellen Ideen, die der Stigmatisierung zugrunde liegen, infrage stellen und für die Gültigkeit der eigenen kulturellen Ideen über „Schlachttiere“ argumentieren. Ferner nutzen die interviewten Schlachthofarbeiter starre Gruppengrenzen, um die Autorität externer Akteure zur Urteilsbildung über Schlachthofarbeit zu delegitimieren. Der Artikel ist innovativ, da er erstmals systematisch die Reaktionsweisen von Schlachthofarbeitern auf moralische Stigmatisierung untersucht.

## Abstract (en)

Slaughterhouse work has a bad reputation and many slaughterhouse workers experience moral stigmatization, although meat consumption is the dominant diet in Western societies. So far, moral stigmatization of slaughterhouse workers has not been analyzed systematically. The article answers the following research questions: Which coping strategies regarding moral stigmatization can be found among slaughterhouse workers and how do these strategies relate to hegemonic narratives about their job? The article answers the research questions using concepts from sociological theories of culture, stigma and *dirty work* and is based on a qualitative content analysis of 13 problem-centered interviews with workers from six German slaughterhouses. The analysis showed that slaughterhouse workers are responding to moral stigmatization by questioning the cultural ideas on which stigmatization is based and by arguing for the validity of their own cultural ideas about “slaughter animals”. The interviewed slaughterhouse workers also use rigid group boundaries to delegitimize the authority of external actors to judge slaughterhouse work. The article is innovative because it systematically analyzes how slaughterhouse workers cope with moral stigmatization.

## Einleitung

Der Umgang westlicher Gesellschaften mit Schlachthofarbeit ist widersprüchlich. Einerseits ist das Schlachten von Tieren Grundlage für den dominanten Ernährungsstil, der auch den Konsum von Fleisch beinhaltet. Andererseits hat Schlachthofarbeit einen schlechten Ruf und Konsument/innen meiden die Konfrontation mit der systematischen Tötung von Tieren. Der Beruf „cattle killer in slaughtering plant“ gehört in den USA zu den Tätigkeiten mit dem geringsten Prestige (Smith und Son 2014). Die in Deutschland sinkenden Zahlen der Auszubildenden zum Fleischer – insbesondere mit der Spezialisierung „Schlachtung“ – können als Indiz für das geringe Prestige des Berufs interpretiert werden (Sebastian 2016, S. 12). Die Gründe für den schlechten Ruf der Schlachthofarbeit sind vielfältig. Neben aktuellen Debatten über die Arbeitssituation im Schlachthof und die Gefahren der Verbreitung von Infektionskrankheiten wie Covid-19 steht die tierethische Dimension des Schlachtens im Mittelpunkt öffentlicher Kritik. Sie bezieht sich sowohl auf die Formen der Behandlung von „Nutztieren“ als auch auf grundlegende Fragen der moralischen Legitimität von Fleischproduktion. Diese Kritik wird nicht nur von der Tierrechtsbewegung und vegan/vegetarisch lebenden Menschen artikuliert sowie medial breit rezipiert (Sebastian 2019). Auch die Mehrheit der europäischen Bevölkerung schätzt den Tierschutz in der Landwirtschaft als nicht ausreichend ein (European Commission 2016).

Dieser Artikel argumentiert, dass Schlachthofarbeit moralisch stigmatisiert wird. Dies betrifft insbesondere diejenigen Schlachthofarbeiter^1^, die unmittelbar in das Töten der Tiere involviert sind. In diesem Artikel werden die Forschungsfragen untersucht, welche Strategien des Umgangs mit moralischer Stigmatisierung sich unter Schlachthofarbeitern finden und wie sie sich zu hegemonialen Erzählungen zu ihrem Beruf verhalten.

In Teil 2 dieses Artikels werden der Forschungsstand und die Forschungslücken im Hinblick auf die Forschungsfragen erörtert. In Teil 3 wird ein theoretischer Rahmen auf Basis soziologischer Theorien zu Stigmatisierung, zu *dirty work* und zu kulturellen Deutungspraxen von Schlachthofarbeitern entwickelt. Dabei wird die Stigmatisierung vor dem Hintergrund der umstrittenen kulturellen Rahmung der Mensch-Tier-Beziehung in westlichen Gesellschaften diskutiert. Der Artikel legt die theoretischen Annahmen zugrunde, dass Schlachthofarbeiter kulturelle Ideen in Bezug auf das Schlachten von Tieren vertreten, die das moralische Stigma reduzieren sollen, und dass die Konstruktion von Gruppenidentität und Abgrenzung zu Außenstehenden hierbei unterstützend wirken. Teil 4 stellt das methodische Vorgehen dar. Zur Beantwortung der Forschungsfragen wurden 13 leitfadengestützte, problemzentrierte Interviews mit Schlachthofarbeitern aus sechs deutschen Schlachthöfen geführt und mit der Qualitativen Inhaltsanalyse ausgewertet. In Teil 5 werden die Ergebnisse der Analyse vorgestellt und in Teil 6 werden diese diskutiert.

## Forschungsstand zu Stigmatisierung, Dirty Work und Schlachthofarbeit

Goffmans (1967) Forschung über Stigmatisierung legte den Grundstein für eine umfangreiche Diskussion über die Ursachen von Stigmatisierung und die unterschiedlichen Stigmareaktionen. Er analysiert, wie sich Gesellschaften ihrer sozialen Normen versichern, indem sie diejenigen sanktionieren, die als außerhalb der Norm betrachtet werden, und wie Stigmatisierte mit öffentlich bekanntem oder unbekanntem Stigma umgehen. Er unterscheidet zwischen Stigmatisierung auf Basis physischer Merkmale (etwa körperlichen Beeinträchtigungen), phylogenetischer Merkmale (etwa Herkunft oder Religionszugehörigkeit) sowie Charakterschwächen, die sich auf Handlungen oder Einstellungen beziehen, die sowohl Formen abweichenden Verhaltens, aber auch explizit als unmoralisch abgelehnte Praxen beinhalten können (ebd., S. 12f.). Soziologische Stigmaforschung diskutiert seither insbesondere die Ursachen und Veränderungsdynamiken von Stigmatisierungsprozessen, Stigmareaktionen und Interaktionsdynamiken in ihrem soziokulturellen und institutionellen Zusammenhang (vgl. Lamont et al. 2013, 2016).

Hughes (1962) prägte den Begriff *dirty work* für Formen von Arbeit mit geringem Prestige. In der Organisations- und Managementforschung wurden unterschiedliche Formen von Stigmatisierung im Kontext von *dirty work* untersucht. Dabei wird *dirty work *als spezifische Form von Stigmatisierung interpretiert, die sich auf die Ausübung von Arbeit bezieht. Ashforth und Kreiner (1999, 2013, 2014) differenzieren unterschiedliche Formen des öffentlich wahrgenommenen Makels, auf dessen Basis eine Arbeit als *dirty *stigmatisiert sein kann. Als „morally tainted work“ (Ashforth und Kreiner 2014, S. 82) gilt Arbeit, die von der gesellschaftlichen Mehrheit als moralisch fragwürdig oder anstößig wahrgenommen wird, etwa Prostitution oder das Ausführen der Todesstrafe. In der umfangreichen Forschungsliteratur zu Stigmatisierung im Allgemeinen sowie zu *dirty work* im Speziellen wurde eine Vielzahl an Reaktionsweisen auf Stigmatisierung auf der Mikroebene diskutiert. Stigmareaktionen unterscheiden sich nach Grund, Art und kulturellem Kontext des Stigmas (vgl. Lamont et al. 2012, S. 44). Häufig wird dabei zwischen aktiven und reaktiven (LeBel 2008; Link und Phelan 2001, S. 378) sowie zwischen individuellen und gruppenbezogenen (Miller und Kaiser 2001, S. 83) Reaktionsweisen unterschieden. Zu der Vielzahl der untersuchten konkreten Reaktionsweisen gehören beispielsweise die Abwehr negativer Attribuierungen (Claire et al. 2016), das Verheimlichen von Stigmata (Goffman 1967; Bursell 2012), das Vermeiden von Konfrontation (Miller und Kaiser 2001; LeBel 2008) oder das Fokussieren auf nicht-stigmatisierende Aspekte des eigenen Lebens oder der Arbeit (Ashforth und Kreiner 2013; Miller und Kaiser 2001). Als besonders relevant für die Entstigmatisierung werden in der sozialwissenschaftlichen Forschungsliteratur aktive, konfrontierende Reaktionen benannt (vgl. Lamont et al. 2013, S. 133; Fleming et al. 2011). Diese umfassen insbesondere Versuche, die kulturellen Ideen der Stigmatisierenden oder das öffentliche Bild der Stigmatisierten zu ändern (Claire et al. 2016; Meisenbach 2010). Hierbei spielen unter anderem sozialer Aktivismus (vgl. LeBel 2008; Lamont et al. 2012, S. 43), das Ziehen vorteilhafter Vergleiche (Meisenbach 2010, S. 280f.) und Aufklärungsarbeit (Fleming et al. 2011), aber auch verschiedene Formen des *reframing* eine Rolle. Beim *reframing* versuchen Akteure, die kulturelle Bedeutung einer Handlung oder Situation zu ändern, um dem Stigma entgegenzuwirken (Ashforth und Kreiner 2013; Miller und Kaiser 2001), etwa indem es als ungerechtes Vorurteil gedeutet wird.

In der Forschungsliteratur zu arbeitsbezogenem Stigma und *dirty work* spiegeln sich die Vielfältigkeit des Stigma-Managements und der empirischen Gegenstände wider. Privates Sicherheitspersonal nutzt beispielsweise unterschiedliche kommunikative Strategien der Identitätsarbeit, um das öffentliche Bild über den eigenen Beruf positiv zu beeinflussen (Brewis und Godfrey 2017). Demgegenüber nutzen viele Sexarbeiterinnen aus dem BDSM-Bereich einer Studie zufolge unter anderem Techniken des Täuschens, Verheimlichens und selektiven Eröffnens des eigenen Berufs sowie der Abgrenzung gegenüber anderen Formen der Sexarbeit (Levey und Pinsky 2014). Schlachthofarbeit spielt in der Forschungsliteratur zu arbeitsbezogenem Stigma bisher eine untergeordnete Rolle. Sie wurde aufgrund der starken physischen Belastung und des Umgangs mit Tod, Blut und Innereien als besonders starke Form von *dirty work* interpretiert (Baran et al. 2016; McCabe und Hamilton 2015). Auch verwandte Tätigkeiten wie die Metzgerei werden in der Literatur als *dirty work* interpretiert (Simpson et al. 2014; Meara 1974). Baran et al. (2016) argumentieren, dass insbesondere die moralische Dimension des Schlachtens die öffentliche Stigmatisierung von Schlachthofarbeit begründe. Die Frage, wie Schlachthofarbeiter mit der Stigmatisierung ihrer Arbeit als unmoralisch umgehen, wurde bisher jedoch nicht untersucht. Zwar wird auch die moralische Dimension der Mensch-Tier-Beziehung im Schlachthof zunehmend zu einem relevanten Gegenstand der Forschung zu Schlachthofarbeit, aber im Mittelpunkt stehen hier der Umgang mit emotionalen Herausforderungen (z. B. McLoughlin 2019; Pachirat 2011) sowie individuelle Sinnstiftungen und Legitimationsstrategien (z. B. Hamilton und Taylor 2013; Sebastian 2014; Cudworth 2011) im Kontext des Tötens von Tieren.

Es mangelt bisher an empirischen und systematischen Analysen der Umgangsweisen von Schlachthofarbeitern mit der moralischen Stigmatisierung des Tötens von Tieren. Da Schlachthofarbeit moralisch stigmatisiert ist, obwohl die gesellschaftliche Mehrheit durch ihr Konsumverhalten die stigmatisierte Arbeit „in Auftrag“ gibt und von dieser profitiert, trägt der Artikel ferner zur soziologischen Stigmaforschung bei, indem er eine Konstellation von Stigmatisierten und Mehrheitsgesellschaft untersucht, die bisher wenig beachtet wurde.

## Kulturelle Rahmungen von Stigmatisierung und Entstigmatisierung der Schlachthofarbeit

Goffman definiert Stigmatisierte als „in unerwünschter Weise anders, als wir es antizipieren“ (1967, S. 13) und macht damit deutlich, dass Stigmata Zuschreibungen, Deutungen und Erwartungshaltungen seitens der Mehrheitsgesellschaft beinhalten. Im Stigma drücken sich kulturelle Ideen über das jeweilige stigmatisierte Merkmal einer Person oder Gruppe und normative Leitbilder der hegemonialen Gruppen aus, die sie gegenüber den Stigmatisierten durchzusetzen versuchen (vgl. Lamont et al. 2016, 2013). Basierend auf diesem Verständnis von Stigmatisierung als Ergebnis kultureller Deutungskonflikte müssen Stigmatisierung und Stigmareaktionen sowie das kollektive Selbstverständnis stigmatisierter Gruppen vor dem Hintergrund der dominanten kulturellen Ideen bzw. der „cultural repertoires“ (Lamont et al. 2013, S. 130) einer Gesellschaft, auf die sich das Stigma bezieht, verstanden werden (vgl. Lamont et al. 2016). Da kulturelle Ideen wandelbar und fragmentiert sowie potenziell umstritten sind (vgl. Pfau-Effinger 2005), können sich stigmatisierte Akteure in den kulturellen Deutungskonflikt über ihr Stigma einbringen. Sie können das Stigma andererseits auch akzeptieren (Meisenbach 2010). Zunehmende Stigmatisierung einer Gruppe kann somit Ausdruck erfolgreicher Durchsetzung einer bestimmten kulturellen Deutung sein und deutet auf einen Wandel der gesellschaftlichen Machtbeziehungen zwischen sozialen Gruppen hin (vgl. Claire et al. 2016, S. 228). Kultureller Wandel kann auch dazu führen, dass die Legitimität sozialer Praxen, die zuvor kulturell nicht oder nur kaum umstritten waren, zunehmend infrage gestellt werden. Dadurch können sich die kulturellen Erwartungen an das Handeln der Akteure ändern und diese müssen einen Umgang mit dem zunehmenden Legitimationsdruck finden.

Stigmatisierte können unterschiedliche Reaktionen zur Entstigmatisierung entwickeln (vgl. LeBel 2008). In demokratischen Gesellschaften besteht eine häufige Reaktion auf Stigmatisierung in dem Versuch der Stigmatisierten, die Sichtweisen und Einstellungen der Stigmatisierenden bzw. der Öffentlichkeit zu ändern (vgl. Claire et al. 2016, S. 223). Bei dieser auch als „identity work“ (Snow und Anderson 1987) bezeichneten Praxis handelt es sich um kollektive Deutungen, bei denen negative (Be‑)Deutungen eines Phänomens durch positive oder neutrale ersetzt werden sollen (vgl. Ashforth und Kreiner 2013, S. 131; Lamont und Mizrachi 2012).^2^

Stigmatisierung wird in diesem Artikel definiert als negative Stereotypisierung sozialer Gruppen (vgl. Link und Phelan 2001; Claire et al. 2016, S. 223). Ein moralisches Stigma wird in Anlehnung an Ashforth und Kreiner (2014) verstanden als die negative Beurteilung spezifischer Charakteristika oder Handlungen einer sozialen Gruppe als explizit unmoralisch. Somit ist der Begriff enger gefasst als die Stigmatisierung von „Charakterschwächen“ im Sinne Goffmans (1967, S. 12f.), da Letztere auch „Willensschwäche, […] tückische und starre Meinungen und Unehrenhaftigkeit“ (ebd.) umfasst, die nicht notwendigerweise als unmoralisch, sondern lediglich als kulturelle Normabweichungen verstanden werden. Im Fall der moralischen Stigmatisierung von Schlachthofarbeit steht insbesondere die Schädigung des Prestiges und der gesellschaftlichen Anerkennung, also des Zugangs zu symbolischen Ressourcen, im Fokus.

Schlachthofarbeit wird in diesem Artikel aufgrund seines geringen Prestiges als *dirty work* interpretiert (vgl. Ashforth und Kreiner 1999, 2013, 2014). *Dirty work* kann als eine spezifische Form von Stigma verstanden werden, das sich auf die Ausübung einer bestimmten Form von Arbeit bezieht, die mit einem geringen Prestige versehen ist und deren Ausübende in unterschiedlicher Weise mit einem stigmatisierenden Makel assoziiert werden (Hughes 1962). Ashforth und Kreiner (2014) differenzieren zwischen physischem, sozialem und moralischem Makel von *dirty work*. Physischer Makel bezieht sich auf körperlich anstrengende Arbeit und das Ausüben von als physisch unangenehm und unsauber empfundenen Tätigkeiten. Sozialer Makel bezieht sich auf den Umgang mit Menschen, die einen geringen sozialen Status besitzen. Moralischer Makel umfasst Arbeit, die kulturell als unmoralisch und anstößig empfunden wird. Schlachthofarbeit kann als „pervasive taint“ (Kreiner et al. 2006) bezeichnet werden, da alle drei Formen des Makels vorliegen: Die Arbeit in Schlachthöfen ist repetitiv, körperlich anstrengend, birgt ein hohes Gesundheits- und Verletzungsrisiko und ist im Zuge der Covid-19-Pandemie auch im Hinblick auf mangelhaften Infektionsschutz kritisiert worden. Zudem gehört die Exposition mit Blut, Innereien und Tod zur Alltagserfahrung von Schlachtern. Der soziale Makel liegt für die festangestellten Arbeitnehmer mir Berufsausbildung in der Zusammenarbeit mit migrantischen Werkvertragsnehmer/innen, da diesen durch die Mehrheitsgesellschaft ein geringerer sozialer Status zugesprochen wird. Sofern Schlachttiere als Gesellschaftsteilnehmer mit geringem sozialen Status verstanden werden, kann auch der Kontakt mit Schlachttieren als Element des sozialen Makels verstanden werden. Im Anschluss an Baran et al. (2016) argumentiert dieser Artikel, dass jedoch die moralische Dimension des Schlachtens die spezifische Qualität ihres Makels bzw. ihres geringen Prestiges ausmacht. Entsprechend liegt dem Artikel die Annahme zugrunde, dass die besondere Qualität des Makels der Schlachthofarbeit aus der systematisch geplanten und massenhaften Verletzung und Tötung von Tieren resultiert.

Schlachthofarbeit ist durch eine spezifische kulturelle Rahmung gesellschaftlicher Beziehungen zu „Schlachttieren“ charakterisiert. Diese Beziehung ist in westlichen Gesellschaften Gegenstand kontroverser Debatten, in deren Zuge die Behandlung von Tieren in der Landwirtschaft von einflussreichen gesellschaftlichen Akteuren – etwa aus Parteien, Medien und sozialen Bewegungen – infrage gestellt wird. Tierrechts- und Tierschutzbewegungen sowie vegan-vegetarische Lebensstile werden populärer und es kommt regelmäßig zu kritischen Medienberichten über die Zustände in Tierställen und Schlachthöfen (Sebastian 2019). Die kulturelle Rahmung der Beziehung zu „Schlachttieren“ ist durch den Widerspruch geprägt, dass die Mehrheit der Konsument/innen westlicher Gesellschaften Fleisch konsumiert, aber der Produktionsprozess von Fleisch kulturell unsichtbar ist (vgl. Pachirat 2011; Hamilton und Taylor 2013). Schlachthofarbeit wird moralisch stigmatisiert, obwohl sie Bedingung für die Ernährungsweise der gesellschaftlichen Mehrheit ist. Beim untersuchten Fall handelt es sich folglich um eine besondere Konstellation von Stigmatisierten und Stigmatisierenden, die aus gleichzeitiger moralischer Infragestellung und persönlicher Unterstützung der diskreditierten Praxis besteht.

Dieser Artikel analysiert, inwiefern Schlachthofarbeiter moralische Stigmatisierung erfahren. Ferner wird analysiert, welche kulturellen Ideen Schlachthofarbeiter in Bezug auf das Schlachten von Tieren vertreten, inwiefern sie sich in den kulturellen Deutungskonflikt über sich und ihre Arbeit einbringen und wie sie versuchen, die kulturellen Ideen über das Schlachten zu ändern. Der Artikel basiert auf der theoretischen Annahme, dass Schlachthofarbeiter moralische Stigmatisierung erfahren und kulturelle Ideen in Bezug auf das Schlachten von Tieren vertreten, die das moralische Stigma reduzieren sollen. Es ist aber auch denkbar, dass Schlachter keine moralische Stigmatisierung erfahren, oder dass sie versuchen, die Konfrontation mit dem Stigma zu vermeiden und sich nicht aktiv in den kulturellen Deutungskonflikt einbringen.

Stigmatisierung und De-Stigmatisierung betreffen soziale Gruppen, die über die Definition der Gruppengrenzen von der nicht-stigmatisierten Außenwelt abgegrenzt werden (Lamont et al. 2013, S. 145f.; Lamont und Mizrachi 2012; Lamont und Molnár 2002). Stigmatisierte Gruppen unterscheiden sich darin, wie stark sie Gruppenidentitäten ausprägen (Lamont et al. 2012). Hohe Gruppenidentifikation kann zu einem kollektiven Empfinden von „cultural sameness“ (Lamont et al. 2013, S. 145) führen. Diese umfasst kollektive, moralische und religiöse Überzeugungen oder gruppenspezifische kulturelle Ideen, die mehr oder weniger mit denen der Mehrheitsgesellschaft übereinstimmen oder konfligieren können. Gruppenidentifikation kann als Ressource im Umgang mit Stigmatisierung dienen, da sie ein Gefühl des Verstanden-Werdens und der geteilten Erfahrungen bietet (Lamont et al. 2013; Ashforth und Kreiner 1999, 2014). Insgesamt scheint im Hinblick auf die Reaktionsweisen von Schlachthofarbeitern mit moralischer Stigmatisierung von Bedeutung, wie stark sich die Beschäftigten mit ihrem Beruf identifizieren. Es ist davon auszugehen, dass gelernte Fleischer ein höheres Maß an Identifikation aufweisen als die mehrheitlich migrantischen Werkvertragsnehmer/innen, die primär aus ökonomischen Zwängen im Schlachthof arbeiten. Der Artikel legt die theoretische Annahme zugrunde, dass die Konstruktion von Gruppenidentität und Abgrenzung zu Außenstehenden unterstützend auf den Umgang von Schlachthofarbeitern mit moralischer Stigmatisierung wirkt.

## Qualitative Interviews mit Schlachthofarbeitern und Feldzugang

Der Artikel basiert auf der Auswertung von 13 Interviews mit Schlachthofarbeitern aus sechs deutschen Schlachthöfen. Der Zugang zum Feld war von anfänglicher Zurückhaltung seitens vieler Unternehmen geprägt. Vier Interviews wurden in zwei Kleinbetrieben, die sich schnell zur Vermittlung von Interviewpartnern bereit erklärten, durchgeführt. Acht weitere Interviews mit Mitarbeitern von vier Großbetrieben – darunter einige Marktführer – waren erst nach einer längeren Phase des Kontaktaufbaus möglich. Es ist wahrscheinlich, dass dies mit der hohen Zahl negativer Medienberichte zur Zeit der Kontaktaufnahme zu tun hatte. Dieser Eindruck wurde durch Gespräche mit Unternehmens- und Betriebsleitungen bestätigt. Der Zugang zu den großen Betrieben wurde durch „door opener“ aus der Fleischindustrie ermöglicht, die zunächst von der Wissenschaftlichkeit des Projekts überzeugt werden mussten. Ein Interview wurde mit einem ehemaligen Schlachter durchgeführt. Die Interviewten waren deutsche Staatsbürger zwischen 18 und 75 Jahren, hatten eine Ausbildung als Fleischer und waren fest angestellt. Dies ist insofern bedeutsam, als dass zum Zeitpunkt der Interviewführung festangestellte, gelernte Fleischer neben prekär beschäftigen, meist osteuropäischen Werkvertragsnehmer/innen nur einen Teil der Schlachthofbelegschaften bildeten. Da für die Tötung der Tiere ein Sachkundenachweis („Schlachtschein“) notwendig ist, kann allerdings davon ausgegangen werden, dass der Anteil festangestellter Schlachthofarbeiter im Bereich der Tötung der Tiere überdurchschnittlich hoch ist. Die Interviews untersuchen somit einen bestimmten Typ Schlachthofarbeiter, der sich durch eine formale Berufsqualifikation, direkte Arbeitsverhältnisse zum Arbeitgeber und einen höheren Kündigungsschutz auszeichnet. Diese „Stammbelegschaften“ sind ein wesentlicher Teil der Schlachthofarbeiter, finden in der öffentlichen Diskussion jedoch weniger Aufmerksamkeit. Es scheint plausibel, dass diese Gruppe an Schlachthofarbeitern auch ein höheres Maß an Gruppenzugehörigkeit und Identifikation mit dem eigenen Beruf aufweist.

Die Interviewpartner in dieser Studie hatten direkte und großteils langjährige Erfahrung in den Bereichen Eintrieb, Betäubung und Tötung von Schweinen und/oder Rindern. Da aus moralischer Perspektive der Behandlung und der Tötung der Tiere größere Relevanz im Vergleich zu deren Zerlegung und Verarbeitung beigemessen wird, erscheint es plausibel, dass die Interviewpartner in besonderem Maß moralische Stigmatisierung erfahren. Ergänzend wurden zum besseren Verständnis der Fleischindustrie Fachmessen besucht und drei explorative Interviews mit Vertreter/innen einer Gewerkschaft, der Berufsgenossenschaft sowie einer Zertifizierungsstelle für den Sachkundenachweis zur Betäubung und Tötung von Tieren geführt.

Ziel der Interviews mit den Schlachthofarbeitern war die Rekonstruktion der subjektiven Sichtweisen und Relevanzsetzungen im Hinblick auf die Erfahrung von und den Umgang mit moralischer Stigmatisierung. Problemzentrierte Interviews sind einerseits durch eine offene Gesprächsführung charakterisiert, durch die den Befragten Raum für thematische Schwerpunktsetzungen gegeben wird, und andererseits durch das Einbringen spezifischer Fragen zum definierten Problembereich (Witzel und Reiter 2012). Die Interviewpartner wurden beispielsweise gefragt, wie sie mit Fremden über ihre Arbeit sprechen oder wie sie die mediale Darstellung der Schlachthofarbeit empfinden. Ein großer Teil des ausgewerteten Materials entstand zudem infolge erzählgenerierender Fragen, aus denen sich zum Teil lange persönliche Narrationen entfalteten, in denen die Interviewten über das Bild des Schlachthofarbeiters in der Öffentlichkeit und ihren Umgang mit diesem sprachen.

Zur Analyse der Daten wurde eine inhaltsstrukturierende Qualitative Inhaltsanalyse, orientiert an Schreier (2012) und Kuckartz (2016), durchgeführt. Diese ermöglicht es, das empirische Material systematisch zu interpretieren und die für die Beantwortung der Forschungsfragen relevanten Aspekte des Materials zu fokussieren. Das Material wurde auf Basis eines Kodierschemas kodiert, das zunächst auf deduktiv entwickelten Kategorien basierte und während des Analyseprozesses überarbeitet und modifiziert wurde. In den induktiven Schritten der Auswertung wurden die spezifischen Ausprägungen der untersuchten kulturellen Deutungen und Stigmareaktionsweisen im Umgang mit moralischer Stigmatisierung rekonstruiert. Dieser iterative Prozess des Modifizierens und „Testens“ des Kodierschemas wurde wiederholt, bis keine weiteren sinnvollen Änderungen möglich waren.

## Reaktionen von Schlachthofarbeitern auf moralische Stigmatisierung

Im Folgenden wird zunächst dargelegt, inwiefern die befragten Schlachthofarbeiter moralische Stigmatisierung erfahren und diese als Problem definieren. Danach wird analysiert, welche Bedeutung die kulturelle Rahmung des Schlachtens für den Umgang von Schlachthofarbeitern mit moralischer Stigmatisierung hat. In einem weiteren Schritt wird analysiert, welche Bedeutung Gruppenzugehörigkeit und -abgrenzung für den Umgang von Schlachthofarbeitern mit moralischer Stigmatisierung haben.

### Erfahrungen moralischer Stigmatisierung von Schlachthofarbeitern

Erfahrungen moralischer Stigmatisierung sind ein bestimmendes Thema der Interviews. Ein Interviewter spricht beispielsweise über den schlechten Ruf von Schlachtern, demgemäß sie als grausam und herzlos im Umgang mit Tieren gelten würden: „Ganz ehrlich, die denken immer, man ist ein Killer.“ (Schlachthof Nr. 1, Arbeiternehmer Nr. 1, Absatz 139, kurz: S1A1: 139) Ein anderer berichtet, Fremde hielten ihn für einen „Tierquäler“ (S5A1). In den Interviews finden sich wiederkehrend Aussagen, falsch und in ungerechter Weise öffentlich dargestellt zu werden, wodurch sich die interviewten Schlachter missverstanden und moralisch kritisiert fühlen. Ein Interviewpartner bezeichnet dies als diskriminierend (S3A1: 176). Ein anderer empfindet seinen Beruf als nachteilig beim Kennenlernen von Frauen, da diese von seinem Beruf abgeschreckt würden (S6A2: 90). Die Mehrheit der Interviewpartner berichtet von Erfahrungen moralischer Stigmatisierung. Nur zwei der Interviewten zeigen keine oder nur sehr geringe Anzeichen moralischer Stigmatisierung (S2A1; S6A1).

In der Regel bleibt das Stigma abstrakt, d. h. unmittelbare Konfrontationen sind seltener als die Vermittlung des Stigmas durch die Medien oder durch das Wissen über das schlechte Image in der Öffentlichkeit. Viele Interviewpartner sprechen daher generalisierend davon, was „die Leute“ ihrer Ansicht nach über Schlachter denken oder wie „die Medien“ über sie berichten. Als Grund für die sinkende Zahl an Auszubildenden nannte ein Interviewpartner etwa das negative Image von Schlachtern in der Öffentlichkeit: „Die sagen ja, […] am besten noch, sagen ja viele, böse Leute oder so.“ (S4A2: 54) Ein anderer fasst die Assoziationen Außenstehender mit Schlachthofarbeit zusammen: „Man meint ja immer so, die Schlachter die sind grausam und auf das Töten aus.“ (Ehemaliger Schlachter, kurz: Ehem.: 22) Die weniger häufigen direkten Stigma-Erfahrungen scheinen allerdings eine größere und nachhaltigere Wirkung auf die Interviewpartner zu haben. Diese direkten Konfrontationen ereignen sich beispielsweise während des Kontakts mit Bekannten und Familienangehörigen oder durch Konfrontation mit Tierrechtsaktionen. Ein Schlachthofarbeiter erinnerte sich an eine Situation, in der er von einer Gruppe Schulkinder, die ihn bei der öffentlichen Erschießung einer aus dem Schlachthof entflohenen Kuh beobachtete, lautstark als „Mörder“ bezeichnet wurde (S1A1: 191). Das brachte ihn dazu, nicht mehr an der Tötung entflohener Tiere teilzunehmen.

### Kulturelle Ideen von Schlachtern als Grundlage für Stigmareaktionen

Die Auswertung der Interviews ergab, dass die Interviewpartner insbesondere im Hinblick auf drei Themenbereich die öffentliche Wahrnehmung der Schlachthofarbeit als moralisch stigmatisierend empfanden. Diese umfassten (1.) die Arbeitspraxen im Schlachthof, (2.) die kulturellen Ideen in Bezug auf „Nutztiere“ und (3.) die Frage der Verantwortung für Mängel im Tierschutz. Im Folgenden wird dargestellt, wie sich die interviewten Schlachter in den kulturellen Deutungskonflikt über diese Aspekte der kulturellen Rahmung ihres Berufs einbringen, um der Stigmatisierung entgegenzuwirken und als unzutreffend erachtete Deutungspraxen zu „korrigieren“.

#### Korrektur kultureller Ideen über Arbeitspraxen im Schlachthof

Die Interviewten benennen unterschiedliche Tätigkeiten im Schlachthof, die aus ihrer Sicht in der Öffentlichkeit zu Unrecht als moralisch fragwürdig kritisiert werden. Diese betreffen vor allem die Behandlung und Tötung der Tiere. Alle Interviewten weisen von sich, Tiere zu misshandeln oder zu quälen. Sie betonen ihr Interesse am Tierwohl und ihre Ablehnung von Tierquälerei. Einige argumentieren, dass schlechte Behandlung von Tieren unwahrscheinlich sei, da dies die Fleischqualität senke und damit ihren eigenen Interessen widerspräche. Viele der Interviewpartner argumentieren, dass Schlachtungen nur dann moralisch relevant seien, wenn den Tieren *unnötiges* Leiden zufügt werde. Sie empfinden Schlachtungen entsprechend als legitim, wenn die Tiere auf eine Weise behandelt werden, die sie als „vernünftig“ oder „ordentlich“ betrachten. Ein Arbeitnehmer erklärt: „Das kann ich gut mit meinem Gewissen vereinbaren, weil die Tiere […] vernünftig behandelt werden und auch tierschutzgerecht behandelt werden.“ (S4A1: 8) Ein anderer erzählt: „Ich habe doch unheimlich gute Beziehungen, auch zu Tieren. Ich würde auch *nie* ein Tier quälen, *nie.* Das muss in Ordnung sein […], muss vernünftig gemacht werden, vernünftig getötet werden und dann […] braucht man sich keine Gedanken machen.“ (S1A1: 97) Kritik an der Ausübung „vernünftiger“ und „ordentlicher“ Schlachtung und damit begründete moralischer Stigmatisierung weisen sie entsprechend als unbegründet zurück. Zudem argumentieren einige Interviewpartner, dass Kritik an den Tierschutzstandards im Schlachthof veraltet sei, da die Schlachtindustrie diese substanziell weiterentwickelt habe. Sie betonen, dass Tierschutzverstöße unwahrscheinlich seien, weil Schlachthöfe durch Veterinärämter und Behörden kontrolliert würden, was die Chance für moralisch relevantes Fehlverhalten unwahrscheinlich mache: „Wir haben zwei Tierärztinnen und […] da brauchst du dir nichts erlauben, die sind so streng.“ (S6A2: 44) Zur Unterstützung ihrer Argumente betonen viele Interviewte die Transparenz der Branche gegenüber der Öffentlichkeit. Während der Interviews boten einige Interviewpartner dem Autor geführte Touren des Betriebs an, „denn wir haben ja auch nichts zu verbergen.“ (S4A2: 120) Ein anderer Schlachter erklärt hingegen, dass seine Firma Besucher/innen nicht erlaube, die Tötung der Tiere zu beobachten, sondern stattdessen ein Video vorführe, „um Missverständnissen […] vorzubeugen.“ (S3A1: 196).

#### Korrektur kultureller Ideen über „Nutztiere“

Die Auswertung der Interviews ergab, dass die Interviewten kulturelle Ideen über die Behandlung und Tötung von „Nutztieren“ identifizieren, die ihren eigenen Sichtweisen widersprechen. Tierethisch motivierte Kritik des Umgangs mit Tieren im Schlachthof wird zwar zumeist nur mit einem kleinen Teil der Bevölkerung assoziiert, aber als relevant und einflussreich genug eingeschätzt, um teils ausführliche Kritiken und Rechtfertigungen seitens der Interviewpartner zu provozieren.

Die Interviewten berufen sich auf tradierte kulturelle Ideen über die alternativlose Notwendigkeit von Fleischkonsum und -produktion. Hierbei spielen unter anderem die Ontologisierung von als „Nutztiere“ definierten Tieren zum Nahrungsmittel eine zentrale Rolle. Ein Interviewpartner erklärt: „Aber, wofür sind sie denn da? *Sind* zum Essen da. Kann einer ja sagen, was er will.“ (S1A1: 87) Ein interviewter Schlachter bringt die objektifizierende Sichtweise auf „Nutztiere“ als Waren und Rohstoffe prägnant auf den Punkt. Ihn fasziniert, dass Schweine „potenzielle Schnitzel sind, anschließend. […] Allein die Produktvielfalt, die aus einem Tier gemacht werden kann. […] Ich finde das total interessant, was man aus einem Tier oder einem Lebewesen alles machen kann.“ (S4A1: 28) Weiter relevant ist die Traditionalisierung der Schlachtung von Tieren als historisch unveränderbar: „Das war schon immer so, dass ein Tier irgendwann unter dem Strich auf dem Teller landet.“ (S2A2: 196) Eine häufige Sichtweise impliziert das Argument, Fleischkonsum sei die einzig mögliche gesunde Ernährungsform. Einer der interviewten Schlachter sagt dazu: „Es *ist* notwendig, weil wir davon leben. […] Es ist einfach wie in der Natur auch. Fressen und gefressen werden ist das System der Natur […]. Eine Kuh *kann* eben nichts anderes als Gras fressen. Und so ist es bei uns auch. Wir müssen tierisches Eiweiß zu uns nehmen, damit wir leben können.“ (Ehem.: 36). Indem die interviewten Schlachthofarbeiter auf diese unterschiedlichen Weisen die Gültigkeit der kulturellen Idee tierlicher Nutzbarkeit betonen, versuchen sie, das moralische Stigma zu delegitimieren, indem dessen kulturelle Grundlage als ungültig erklärt wird.

#### Korrekturen der Verantwortungszuweisungen für Mängel im Tierschutz

Die interviewten Schlachter vertreten die Sichtweise, dass Schlachtungen nur dann moralisch relevant sind, wenn sie nicht „korrekt“ ausgeführt werden, d. h. wenn der Tierschutz nicht ausreichend gewahrt wird. Da dies aus Sicht der Interviewpartner nur in Ausnahmen vorkommt, sei der normale Betrieb eines Schlachthofs moralisch legitim. Der Vorwurf systematisch schlechter oder grausamer Behandlung von Tieren in der Fleischindustrie wird als typisch, aber ungerecht und unhaltbar eingeschätzt. Die häufigste Reaktion besteht jedoch darin, die Verantwortung für die Bedingungen in der Fleischproduktion auf die Konsument/innen zu übertragen. Während mit dem Verweis auf die Kontrolle durch Behörden und Veterinär/innen die Möglichkeit moralisch fragwürdiger Handlungen eher verneint wird, basiert die Verantwortungsverschiebung auf Konsumierende auf der Anerkennung bestimmter Probleme wie Massentierhaltung oder Fehler bei der Betäubung. Viele der interviewten Schlachter argumentieren, die Verhältnisse in der industriellen Tierhaltung und Schlachtung seien das Resultat des Preisdrucks und des Wunsches der Konsumierenden nach günstigen Fleischprodukten. Ein Interviewpartner erklärt: „Wenn da drei Hähnchen für fünf Euro liegen. […] Ein Pfund Gehacktes für eins-neunundneunzig. Ja, was erwartest du denn für Verhältnisse in den Ställen? Wie soll das vonstattengehen? Dass die Schweine alle draußen rumlaufen und lieb gestreichelt werden? Nein, das funktioniert so nicht. Das ist eine Geldsache. […] Der Verbraucher *will* das so.“ (S3A1: 168) Durch die Verschiebung von Verantwortung für Mängel im Tierschutz auf Konsument/innen entkräften die Interviewpartner auch die moralische Stigmatisierung, die dem Argument nach ebenso die Mehrheit der Gesellschaft betreffen müsse. Der Verweis auf die kulturelle Dominanz von Fleischkonsum diente hier folglich als wesentliche Grundlage der Abwehr moralischer Stigmatisierung.

Die Auswertung der Interviews ergab, dass die interviewten Schlachter auf moralische Stigmatisierung vor allem durch eigene, stigmakorrigierende Deutungspraxen reagieren. Sie stellen die Gültigkeit derjenigen kulturellen Ideen infrage, denen gemäß ihr Beruf moralisch fragwürdig oder abzulehnen sei, und ersetzen diese durch eigene kulturelle Ideen, die sie für plausibler und legitimer halten. Ferner nutzen sie ihre kulturellen Ideen über sich und ihre Arbeit, um das ihrem Beruf anhaftende moralische Stigma zu entkräften. Sie kritisieren Vorstellungen, denen gemäß Schlachthofarbeiter grausam gegenüber Tieren seien, und argumentieren für die moralische Legitimität der Behandlung von Nutztieren als Rohstoffe für Fleischproduktion. Verantwortung für die Zustände in der industriellen Tierproduktion und etwaige Tierschutzmängel im Schlachthof verorten sie vor allem bei den Konsument/innen von Fleischprodukten.

### Gruppenidentität und -abgrenzung beim Umgang mit moralischer Stigmatisierung

Gruppenzugehörigkeit spielt in zweierlei Hinsicht eine wesentliche Rolle beim Umgang von Schlachthofarbeitern mit moralischer Stigmatisierung. Dies betrifft die Aberkennung der Deutungsautorität stigmatisierender Gruppen sowie stigmakorrigierende, positive Selbstbeschreibungen.

#### Aberkennung der Deutungsautorität stigmatisierender Gruppen

Die interviewten Schlachter identifizieren mehrere soziale Gruppen, denen sie eine zentrale Rolle bei ihrer Stigmatisierung zusprechen. Diese umfassen Medien, Vegetarier/innen bzw. Veganer/innen und Tierschützer/innen. Neben der Assoziation mit bestimmten Gruppen verknüpfen die Interviewpartner moralische Stigmatisierung auch mit den vorherrschenden Ansichten in der Öffentlichkeit. Die Interviewpartner wehren die als stigmatisierend empfundenen Urteile dieser Gruppen ab, indem ihnen die Autorität und Legitimation abgesprochen wird, ein Urteil über das Schlachten fällen zu können. Diese Form der Stigmaabwehr beinhaltet die „Gegen-Stigmatisierung“ der betreffenden Gruppen als uninformiert, verallgemeinernd, weltfremd, übertreibend oder als unaufrichtig und unehrlich.

Medien werden am häufigsten als stigmatisierende Außenstehende benannt. Sie werden als uninformiert, übertreibend und generalisierend beschrieben. So beschwert sich ein Schlachter, Missstände in einzelnen Schlachthöfen würden auf alle Betriebe zurückfallen (S6A2: 88). Ein interviewter Arbeiter beschwert sich, durch die Medien werde „die Branche ja noch schlechter gemacht, wie sie überhaupt ist. Medien können ja viel kaputt machen.“ (S3A1: 162) Der wichtigste Kritikpunkt ist, dass der mediale Fokus auf Tierschutz-Probleme nicht die Realität des Schlachtens widerspiegle. Wenn vereinzelt positive Beispiele für Medienberichte genannt werden, sind dies Berichte, die dem Narrativ der „Insider-Sicht“ der Schlachter näher folgen und keine moralische Kritik des Schlachtens beinhalten. Medien werden vor allem als „Verstärker“ des Stigmas verstanden, der die stigmatisierenden Ideen einer breiten Öffentlichkeit vermittelt.

Auch Tierschützer/innen, Vegetarier/innen und Veganer/innen werden als uninformiert, verallgemeinernd und übertreibend kritisiert. Ihre Darstellungen der Fleischindustrie werden als falsch und irreführend eingeschätzt, weil sie der als authentisch und korrekt eingeschätzten eigenen „Innen-Ansicht“ der Arbeitsabläufe und Zustände im Schlachthof widersprechen. Die Einschätzungen von Protestaktionen reichen von grundsätzlicher Toleranz friedlicher Proteste bis zur Darstellung von Aktivist/innen als „Verrückte“ (S5A1: 309), die „auf einem ganz verkehrten Mond“ (S3A1: 174) leben. Der Vorwurf der unzulässigen Verallgemeinerung bezieht sich beispielsweise auf heimliche Recherchen von Tierrechtsorganisationen. Kritik an einzelnen Betrieben würde in unzulässiger Weise auf alle Schlachthöfe bezogen: „[Die] ziehen einfach alles in Grund und Boden rein und machen alles schlecht.“ (S2A2: 190) Zu einer solchen grundlegenden Kritik, so ein weiterer Schlachthofarbeiter, „hätte man ja nur ein Recht zu, wenn man wirklich Ansätze *hätte* zur Kritik und die sehe ich wirklich nicht gegeben.“ (S4A2: 266).

Viele stigmatisierende Sichtweisen auf Schlachthofarbeit werden jedoch keiner konkreten Akteursgruppe zugeordnet, sondern einer abstrakten Öffentlichkeit. Dieser wird ein Mangel an Wissen über die tatsächlichen Verhältnisse im Schlachthof, wie sie sich aus Sicht der Interviewten darstellen, attestiert. Viele der interviewten Schlachthofarbeiter nehmen an, dass Konsument/innen mit dem Thema Fleischkonsum in einer Form bewussten Ignorierens umgehen, indem sie Fleischprodukte kaufen, ohne sich für die Innenwelt des Schlachthofs zu interessieren. Einer sagt: „*Jeder* isst Fleisch, aber keiner weiß wirklich, wie es auf den Teller kommt.“ (S6A2: 38) Häufig wird kritisiert, Außenstehende würden die Untrennbarkeit von Fleischproduktion und Tiertötungen in einer naiven Weise ignorieren. Ein Schlachter erklärt: „Leider muss der [Schlachter] das Tier töten, denn du kannst es nicht lebend in den Döner reintun.“ (S5A1: 103) Die Öffentlichkeit wird durch die Interviewpartner als von der tierhaltenden Landwirtschaft entkoppelt wahrgenommen. Hieraus folgt aus Sicht der Interviewten auch eine falsche und unangemessene Beziehung und Einstellung zu „Nutztieren“. Insbesondere durch diese Aussagen wird deutlich, dass die Interviewpartner zuweilen die widersprüchliche kulturelle Rahmung der Fleischproduktion für ihr Stigmamanagement, d. h. zur eigenen Entlastung, nutzen. Die Interviewpartner betonen, dass die stigmatisierende Öffentlichkeit durch ihr (mehrheitliches) Konsumverhalten zwar den Auftrag zur Produktion von Fleisch gebe, die moralische Last jedoch gleichsam auf den Schlachthofarbeiter „abladen“ würde.

Diese Beispiele verdeutlichen die Relevanz der Konstellation von Insidern und Outsidern beim Umgang mit Stigmatisierung. Aus den jeweiligen Positionen ergeben sich unterschiedliche, oft gegensätzliche Deutungspraxen, die konflikthaft aufeinandertreffen können. Dies illustriert auch die Aussage eines Schlachthofarbeiters, der sich im Kindergarten seines Sohnes rechtfertigen musste, nachdem er diesen mit in den Schlachtbetrieb genommen hatte. Der Sohn hatte danach den Schlachtvorgang an anderen Kindern nachgespielt: „Also da hat er alle in der Reihe dargestellt, so hintereinander, und dann hat er sie in die Tötebucht zusammentrieben, hat sie erschossen und ihnen den Hals abgeschnitten und das haben die Erzieherinnen nicht lustig gefunden.“ (S5A1: 97) In dem sich nach diesem Ereignis entwickelnden Streitgespräch mit den Erzieher/innen lehnte der interviewte Schlachter die Deutungen der Erzieher/innen deutlich ab und empfand diese als nicht nachvollziehbar. Hier wird deutlich, wie Praxen, die dem interviewten Schlachter als normal und unbedeutend erscheinen, durch Außenstehende als empörend und unmoralisch interpretiert werden können.

#### Stigmakorrigierende, positive Selbstbeschreibungen

An die Stelle der abgelehnten Fremdbeschreibungen durch stigmatisierende Außenstehende setzen die interviewten Schlachter positive Eigendarstellungen, die das moralische Stigma entkräften sollen. Diese Selbstbeschreibungen zielen nicht nur auf die Restitution des persönlichen Rufes, sondern vor allem des öffentlichen Ansehens des Berufs als Schlachter. Da sich moralische Stigmatisierung individuell als Erfahrungen über die Zugehörigkeit zu einer stigmatisierten Gruppe ausdrückt, reichen stigmakorrigierende Selbstbeschreibungen in ihrem Bedeutungsgehalt über die rein individuelle Identitätskonstruktion hinaus. Insbesondere die als typisch eingeschätzte Darstellung als herzlose und aggressive Menschen, die Freude am Quälen von Tieren haben, wird von den interviewten Schlachtern als entwürdigend und beleidigend empfunden. Ein Schlachter betont: „Es ist auch nicht, dass ich hier irgendwie sadistisch veranlagt […] bin. Ich bin tierlieb.“ (S5A2: 55) Demgegenüber betonen einige interviewte Schlachthofarbeiter, sie seien ruhige Menschen, die zu tiefen Emotionen fähig seien. Ein Interviewpartner macht mehrfach während des Interviews deutlich, seine starke Tierliebe und sein Beruf als Schlachter widersprächen sich nicht: „Ich bin unheimlich *tierlieb*, also das mag kein Mensch glauben, aber es ist so. Ich mag Pferde gerne, ich mag Tiere *richtig* gerne. Ich bin *wirklich* tierlieb. Nur, es ist mein Job.“ (S1A1: 107) Die Darstellung, Schlachter übten ihre Arbeit in einem „Blutrausch“ aus, lehnt er ab: „Wenn ich das schon höre, „Blutrausch“, so etwas gibt es überhaupt gar nicht. […] Das ist alles dummes Zeug. Und das ist immer das Schlimme, diese Erfindungen von den anderen Leuten. Dieses Dramatisch-Machen immer, das ist nicht in Ordnung.“ (ebd.: 313) Ein weiterer sagt: „Auch einer, der schlachtet, *kann* Tiere lieben, mögen oder gernhaben. Ich gehöre zu denen.“ (Ehem.: 30).

Alle Interviewpartner machen Gebrauch von vorteilhaften Vergleichen zwischen der Schlachthofarbeit und anderen Tätigkeiten. Dadurch erhöhen sie entweder den Status der Schlachtarbeit durch den Vergleich mit moralisch nicht stigmatisierten Aktivitäten, oder sie grenzen sich von Tätigkeiten ab, die in ihren Augen moralisch verwerflicher sind. Vergleiche werden beispielsweise hergestellt zu Tischlern, Schustern oder der Medizin, aber auch zu Kampfkunst, Autobau oder Stahlarbeit. So berichtet ein Interviewpartner: „Das ist doch nichts anderes als wie ein SCHUSTER jetzt, ein Schlachter, oder? Ja ist so. Ich meine, die berichten ja auch nicht großartig „Man, was hat der für große Löcher auf den Schuhen gehabt“. Ist doch so. Beim Schlachter, wenn da was ist: „Oh!“ [gespielte Empörung]“ (S1A1: 311). Ein anderer erklärt: „Ja gut, ist halt, du arbeitest auf dem Bau, ist genauso als wenn du eine Wand hochziehst. […] Ne, oder oder andere Tätigkeiten machst oder so. Genauso normal ist das für mich.“ (S3A1: 150).

Ebenso betonen einige der interviewten Schlachter ihre handwerklichen Fertigkeiten als Fleischer, die sie zwar im Schlachthof nicht ausüben, aber die zu ihrem Beruf gehören – insbesondere die Herstellung von Wurst. Auch dies kann als Versuch interpretiert werden, das Ansehen des Berufs zu verbessern. Einige Interviewpartner distanzieren sich darüber hinaus beispielsweise von Großwildjagden, von bestimmten Aspekten der Fleischproduktion wie Halal-Schlachtungen und langen Tiertransporten oder sie kritisieren „schwarze Schafe“ (Ehem.: 78) in der Branche, die wegen Tierschutzverstößen in der Kritik stehen. So kritisiert ein Interviewpartner lange Schlachttransporte als das relevantere Problem: „Solange die Tiere noch von Polen nach Israel fahren, braucht mir keiner etwas von Tierwohl erzählen.“ (S5A1: 343) Der Interviewpartner nutzt ferner das Thema des rituellen Schlachtens zur Abgrenzung innerhalb der eigenen Branche: „Wenn die Türken ihren Feiertag haben, ihren Opfertag, da interessiert es keinen Menschen. Da sage ich immer, „die dürfen denen lebendig den Hals abschneiden, […] das hat mit Tierschutz auch was zu tun“. Und wenn du dann einem der Tiere da mit dem Stupfer eine gibst, das ist wie Mord.“ (S5A1: 125).

Wie dargestellt werden konnte, nutzen die interviewten Schlachthofarbeiter die eigene Gruppenzugehörigkeit als Ressource im Umgang mit moralischer Stigmatisierung. Von zentraler Bedeutung ist hierfür die Konstellation von Insidern und Outsidern im Schlachthof. Die Interviewten delegitimieren Urteile und Ideen Außenstehender über Schlachthofarbeit auf Basis ihres unterstellten mangelnden Wissens über Abläufe und Zustände im Schlachthof. Die als unwahr und falsch eingeschätzten kollektiven Fremdzuschreibungen durch Outsider werden durch zahlreiche Beispiele von den Befragten illustriert und durch positive (Selbst‑)Beschreibungen der Schlachthofarbeiter ersetzt.

## Diskussion und Ausblick

### Diskussion der Forschungsergebnisse

Die von den untersuchten Schlachtern gemachten Erfahrungen moralischer Stigmatisierung beziehen sich vor allem auf das negative Image des Schlachtberufs als moralisch fragwürdig. Dass Erfahrungen von Stigmatisierung vor allem abstrakt und seltener in Form direkter Konfrontationen gemacht werden, kann als Indikator für die gesellschaftliche Relevanz kultureller Ideen, die dem Schlachten kritisch gegenüberstehen, interpretiert werden. Das Wissen um die dominanten kulturellen Ideen in der Gesellschaft wirkt stabilisierend für die Stigmatisierung, denn selbst diejenigen Schlachter, die nicht viel über direkte Stigmaerfahrungen sprechen, scheinen genau zu wissen, was Außenstehende über sie und ihre Arbeit denken.

Die Reaktionen der Schlachthofarbeiter auf ihre moralische Stigmatisierung können als Beitrag zum kulturellen Deutungskonflikt um die Gültigkeit kultureller Ideen gegenüber Schlachtungen, „Schlachttieren“ und Schlachtern verstanden werden. Sie bilden eine soziale Wirklichkeit ab, in der Schlachthofarbeit umstritten, aber der kulturelle Deutungskonflikt aus Sicht der Schlachthofarbeiter noch nicht „verloren“ ist. Die Interviewten ergeben sich nicht ihrem Stigma, sondern widersetzen sich ihm. Durch das *reframing* der eigenen Arbeitspraxen und der Identität versuchen die Interviewten, die Stigmatisierung abzuwehren. Aus dem empirischen Datenmaterial wird jedoch nicht deutlich, inwiefern sich die Interviewpartner auch öffentlich in den Konflikt einbringen.

Ferner kann die Analyse zeigen, dass Gruppenidentifikation und -abgrenzung wichtige Ressourcen für den Umgang der interviewten Schlachter mit moralischer Stigmatisierung sind. Negative Zuschreibungen – etwa als „herzlose Tierquäler“ – ersetzen sie durch positive Selbstbeschreibung und ein kollektives Berufsethos, demgemäß Schlachten nur dann moralisch relevant ist, wenn es nicht sachgemäß ausgeführt wird. Darüber hinaus wehren sie Kritik durch Außenstehende mit dem Verweis ab, dass für die korrekte Einschätzung und moralische Einordnung der Schlachtarbeit Wissen über die „Innenwelt“ des Schlachthofs notwendig ist, das nur Insider haben können. Diese dualistische Konstruktion der richtigen, legitimen Sicht von Insidern und der falschen, illegitimen Sicht der Außenstehenden ist von zentraler Relevanz für die Abwehr moralischer Stigmatisierung. Auffallend ist, dass die Interviewpartner in ihren Darstellungen dieser „richtigen“, auf Insiderwissen basierenden Behandlungsweisen von Tieren meist vage bleiben. Bedeutsamer scheint für die Interviewpartner die grundsätzliche Delegitimierung von als moralisch stigmatisierend empfundenen Ideen externer Kritiker/innen.

Die Auswertungsergebnisse bestätigen die Relevanz hoher Gruppenidentifikation von Schlachthofarbeitern (vgl. Thompson 1983, S. 223; Ackroyd und Crowdy 1990). Als pauschalisierend und stigmatisierend empfundene Ideen scheinen diese nicht nur als Angriff auf ihre persönliche moralische Integrität, sondern auf die ihres Berufs insgesamt zu interpretieren. Der Artikel unterstützt ferner die Relevanz von „cultural sameness“ (Lamont et al. 2013, S. 145) als Ressource zur Stigmaabwehr. Die geteilten kulturellen Ideen über Schlachthofarbeit und die Betonung der Bedeutung von Insiderwissen, das nur innerhalb der eigenen Gruppe verortet wird, dienen den interviewten Schlachthofarbeitern als Schutz gegen die äußeren Infragestellungen ihrer moralischen Integrität. Anzeichen für Versuche der Auflösung der Gruppengrenzen und der Betonung von Gleichheit zwischen Stigmatisierten und Stigmatisierenden (vgl. Claire et al. 2016, S. 225) lassen sich lediglich in der Betonung der eigenen Normalität finden. Diese Versuche der Normalisierung zielen jedoch weniger auf die Infragestellung der Insider-Outsider-Dualität, sondern dienen vielmehr der Abwehr negativer Zuschreibungen gegenüber der eigenen Gruppe und der Wiederherstellung beschädigten Prestiges der Schlachthofarbeit.

Auffallend ist zudem die weitgehende Abwesenheit von Berichten der Interviewpartner über „reaktive“ Umgangsweisen mit moralischer Stigmatisierung, wie Täuschung, Verbergen oder Selbstisolation (vgl. Goffman 1967). Das gezeigte Stigmamanagement der Interviewten war vielmehr motiviert von der Wiederherstellung des beschädigten Rufs. Hierfür sollen im Folgenden zwei mögliche Gründe diskutiert werden: Erstens könnte die Dominanz aktiver Stigmareaktionen durch die besonderen Umstände der Datenerhebung verstärkt worden sein. Zunächst schien der Interviewer von den Interviewpartnern als potenzieller externer Kritiker gesehen zu werden, von dem weitere moralische Stigmatisierung zu befürchten war. Viele Interviews begannen entsprechend mit Skepsis seitens der Interviewten. Mehrfach führte die Eingangsfrage („Können Sie mir bitte einen normalen Arbeitstag beschreiben?“) unmittelbar zu einer Verteidigung der Tierschutz-Standards im Betrieb, ohne dass dieses Thema durch den Interviewer angesprochen wurde. Die anfänglichen Spannungen wichen allerdings in allen Interviews nach kurzer Zeit und die Rolle des Interviewers wechselte in den Augen der Interviewten danach offenbar zum potenziellen Unterstützer. Viele Interviewte schienen die Möglichkeit zu begrüßen, ihre Sicht der Dinge ausführlich darlegen zu können. Die direkte Form des Interviews, die ungewöhnliche Möglichkeit, gehört zu werden, und die Anwesenheit eines interessierten Außenseiters könnten das Vorkommen aktiver Reaktionsweisen verstärkt haben.

Zweitens haben alle Interviewpartner eine Ausbildung zum Fleischer absolviert. Es scheint plausibel, dass hiermit auch ein höheres Maß an Identifikation mit dem eigenen Beruf einhergeht und dass aus dieser eine erhöhte Motivation zum aktiven Umgang mit Stigmatisierung resultiert. Hierin unterscheiden sich die Interviewpartner vermutlich von Werkvertragsnehmenden und Leiharbeitenden, die in der Regel keine Ausbildung als Fleischer durchlaufen haben. Ihre Identifikation mit dem Beruf als Schlachthofarbeiter/innen ist vermutlich geringer, wodurch reaktive Umgangsweisen mit moralischer Stigmatisierung überwiegen könnten. Dieser mögliche Zusammenhang kann jedoch auf Basis der empirischen Daten nicht weiter analysiert werden.

### Zusammenfassung der Auswertungsergebnisse

Die meisten der für diesen Artikel interviewten Schlachthofarbeiter machen Erfahrungen moralischer Stigmatisierung aufgrund ihrer Arbeit im Schlachthof. Anstatt die moralische Stigmatisierung ihrer Arbeit als unabänderlich hinzunehmen, konfrontieren die Interviewpartner die Stigmatisierung diskursiv mit Gegendarstellungen und der Delegitimierung von Kritik. Sie lehnen gängige, als stigmatisierend empfundene kulturelle Ideen über sich und ihre Arbeit ab und versuchen, deren kulturelle Gültigkeit zu minimieren. Damit „korrigieren“ sie als falsch und illegitim betrachtete Sichtweisen und Vorstellungen über bestimmte Arbeitspraxen im Schlachthof, kulturelle Ideen in der Öffentlichkeit über „Nutztiere“ sowie hinsichtlich der Frage nach Verantwortung für mögliche Mängel im Tierschutz. Fraglich ist jedoch, ob die Deutungspraxen der interviewten Schlachter in der Lage sind, die öffentliche Meinung zu ändern.

Ferner kann gezeigt werden, dass Gruppenidentifikation und -abgrenzung von Bedeutung für den Umgang der interviewten Schlachthofarbeiter mit moralischer Stigmatisierung ist. Als stigmatisierend empfundene Beschreibungen ihrer Identität ersetzen diese durch positive Selbstbeschreibungen. Die Autorität außenstehender Akteure zum Fällen von Werturteilen und zur Kritik stellen sie infrage. Ein zentrales Argument zur Abwehr moralischer Stigmatisierung ist, dass nur Schlachter authentisches und korrektes Insider-Wissen über die Innenwelt des Schlachthofs haben, wodurch nur sie selbst legitimiert sind, die Vorgänge im Schlachthof adäquat beurteilen zu können. Moralische Stigmatisierung wird entsprechend als Resultat von Unwissenheit, Naivität oder Übertreibung abgelehnt.

### Beiträge zur soziologischen Theorie und weitere Forschungsmöglichkeiten

Dieser Artikel leistet einen wichtigen Beitrag zum Verständnis darüber, wie Schlachthofarbeiter sich und ihre Arbeit im Kontext moralischer Stigmatisierung wahrnehmen. Er analysiert die Ursachen für und Reaktionen auf moralisches Stigma von Schlachthofarbeit vor dem Hintergrund eines gegenwärtigen, kulturellen Deutungskonflikts in der Gesellschaft über legitime Formen der Behandlung von Tieren. Damit untersucht er einen besonderen Fall von Stigmatisierung: Da Schlachthofarbeit moralisch stigmatisiert ist, obwohl die gesellschaftliche Mehrheit durch ihr Konsumverhalten die stigmatisierte Arbeit „in Auftrag“ gibt und von dieser profitiert, nimmt der Artikel einen Typ der Stigmatisierung in den Blick, der durch eine spezifische Konstellation von Stigmatisierten und Mehrheitsgesellschaft charakterisiert ist. Wie gezeigt wurde, können moralisch Stigmatisierte in einer solchen Konstellation diese Widersprüche für ihr Stigmamanagement nutzen, indem sie auf die als inkonsistent wahrgenommene Legitimationsgrundlage des Stigmas verweisen. Hieran schließt die Frage an, inwiefern mit der moralischen Stigmatisierung von Schlachthofarbeitern auch eine gesellschaftliche Entlastungsfunktion gegenüber dem Fleischkonsum einhergeht, da die moralische Stigmatisierung von Schlachthofarbeit in einer mehrheitlich fleischkonsumierenden Gesellschaft auch als Schuld- und Verantwortungsdiffussion zugunsten der Konsument/innen und zulasten der Schlachthofarbeiter interpretiert werden kann.

Durch die spezifischen Umstände des Feldzugangs ergeben sich unausweichliche methodische Leerstellen des Artikels. Vergleichende Analysen hinsichtlich der Dimensionen Beschäftigungstyp, Schlachttierart und Schlachtmethoden waren nicht möglich. Hier können zukünftige Forschungsarbeiten ansetzen. Schlachthofarbeit ist ein vielversprechendes soziologisches Forschungsfeld. Sie bietet nicht nur Gelegenheit zu Forschungen über oftmals prekäre Industriearbeit, sondern ermöglicht auch die Analyse eines sich gegenwärtig vollziehenden kulturellen Wandels der Mensch-Tier-Beziehungen. Da das Schlachten von Tieren zunehmend zum Gegenstand kontroverser Debatten wird, scheint es wichtig, dass auch die Soziologie sich vermehrt den kulturellen Deutungskonflikten über die legitimen Mensch-Tier-Beziehungen widmet.

## Footnotes

1 Im Folgenden wird ausschließlich die männliche Form genutzt, da Eintrieb, Betäubung und Tötung von Tieren in den für diesen Artikel untersuchten Schlachthöfen fast ausschließlich durch Männer ausgeübt werden.

2 Zudem kann Stigmatisierung vielfältige psychische Belastungen auslösen, die aus der gesellschaftlichen Ablehnung und Abwertung der Stigmatisierten resultieren (vgl. z. B. Herek et al. 2013; Alimoradi et al. 2020). Diese psychologische Dimension moralischer Stigmatisierung kann hier jedoch nicht vertiefend behandelt werden.
